# Prenatal Exposure to Phthalate Esters and Behavioral Syndromes in Children at 8 Years of Age: Taiwan Maternal and Infant Cohort Study

**DOI:** 10.1289/ehp.1307154

**Published:** 2014-10-03

**Authors:** Yin-Ju Lien, Hsiu-Ying Ku, Pen-Hua Su, Suh-Jen Chen, Hsiao-Yen Chen, Pao-Chi Liao, Wei-J. Chen, Shu-Li Wang

**Affiliations:** 1 Department of Health Promotion and Health Education, National Taiwan Normal University, Taipei, Taiwan; 2 Institute of Epidemiology and Preventive Medicine, College of Public Health, National Taiwan University, Taipei, Taiwan; 3 Graduate Institute of Life Science, National Defense Medical Center, Taipei, Taiwan; 4 Division of Environmental Health & Occupational Medicine, National Health Research Institutes, Miaoli, Taiwan; 5 Department of Pediatrics, Chung Shan Medical University, Taichung, Taiwan; 6 Genetic Consultation Center, Chung Shang Medical University Hospital, Taichung, Taiwan; 7 School of Nursing, Chung Shang Medical University, Taichung, Taiwan; 8 Department of Environmental and Occupational Health, College of Medicine, National Cheng Kung University, Tainan, Taiwan; 9 Department of Public Health, National Defense Medical Center, Taipei, Taiwan; * These authors contributed equally to this work.

## Abstract

Background: Few studies have shown an association between prenatal phthalate exposure and adverse effects on neurodevelopment and behavior in young children.

Objectives: We aimed to assess the relationship between prenatal exposure to phthalate esters and behavior syndromes in children at 8 years of age.

Methods: A total of 122 mother–child pairs from the general population in central Taiwan were studied from 2000 to 2009. Mono-methyl phthalate (MMP), mono-ethyl phthalate (MEP), mono-butyl phthalate (MBP), mono-benzyl phthalate (MBzP), and three di-(2-ethylhexyl) phthalate (DEHP) metabolites—mono-2-ethylhexyl, mono-2-ethyl-5-hydroxyhexyl, and mono-2-ethyl-5-oxohexyl phthalates (MEHP, MEHHP, and MEOHP)—were measured in maternal urine collected during the third trimester of pregnancy using liquid chromatography–electrospray ionization–tandem mass spectrometry. Behavioral syndromes of children at 8 years of age were evaluated using the Child Behavior Checklist (CBCL). Associations between log_10_-transformed creatinine-corrected phthalate concentrations and standardized scores of the CBCL were estimated using linear regression models or multinomial logistic regressions with adjustments for potential confounders.

Results: Externalizing problem scores were significantly higher in association with a 1-unit increase in log_10_-transformed creatinine-corrected concentrations of maternal MBP (β = 4.29; 95% CI: 0.59, 7.99), MEOHP (β = 3.74; 95% CI: 1.33, 6.15), and MEHP (β = 4.28 ; 95% CI: 0.03, 8.26) after adjusting for the child’s sex, intelligence, and family income. Meanwhile, MBP and MEOHP were significantly associated with Delinquent Behavior and Aggressive Behavior scores. The same pattern was found for borderline and/or clinical ranges.

Conclusions: Our findings suggest positive associations between maternal DEHP and dibutyl phthalate (DBP) exposure and externalizing domain behavior problems in 8-year-old children.

Citation: Lien YJ, Ku HY, Su PH, Chen SJ, Chen HY, Liao PC, Chen WJ, Wang SL. 2015. Prenatal exposure to phthalate esters and behavioral syndromes in children at 8 years of age: Taiwan Maternal and Infant Cohort Study. Environ Health Perspect 123:95–100; http://dx.doi.org/10.1289/ehp.1307154

## Introduction

Phthalate esters are a family of chemicals that are widely used in daily lives, including food packaging, children’s toys, and building materials. For instance, di-(2-ethylhexyl) phthalate (DEHP) can be found in food containers, butylbenzyl phthalate (BBzP) in vinyl flooring and wall covering, and di-butyl phthalate (DBP) in personal care products (Duty et al. 2005; Schettler 2006). Due to widespread contamination, these chemicals can enter the human body through daily ingestion and inhalation. In addition, personal care products are likely the predominant source of diethyl phthalate (DEP), dimethyl phthalate (DMP), and di-*n*-butyl phthalate (DnBP) (Just et al. 2010). Medically related products contaminated with phthalates are another source of exposure (Hernández-Díaz et al. 2009). Phthalates have been identified in the urine samples of all age groups (Chen et al. 2008).

There is an emerging public health concern that the widespread use of phthalates may affect brain development and children’s behavior. Engel et al. (2010) have reported an association between prenatal low-molecular-weight (LMW) phthalate exposure [the sum of DMP, DEP, DBP, and di-isobutyl phthalate (DiBP)] and poorer scores on aggression, attention problems and depression in children between 4 and 9 years. Another study using the Child Behavior Checklist (CBCL) reported a positive association between prenatal DnBP and BBzP exposure and clinically withdrawn behavior in 3-year-old children (Whyatt et al. 2012). One cross-sectional study revealed that exposure to DEHP might be associated with hyperactivity and attention deficit/hyperactivity disorder (ADHD) symptoms in school-age children (Kim et al. 2009). These studies showed mixed findings in terms of particular phthalates and internalized (e.g., withdrawal, depression) or externalized behavior problems (e.g., delinquent behavior and aggression). Accordingly, more prospective follow-up studies are needed to clarify whether exposures to particular phthalates during the fetal period are associated with distinct behavioral profiles later in childhood.

Animal studies have also indicated that phthalate exposures may affect the development of the central nervous system and brain function: Perinatal exposure to DEHP affected the development of coordinated movements in mice offspring during early postnatal development (Tanaka 2002). Perinatal exposure to DBP induced cell apoptosis in hippocampal neurons and impaired the spatial learning and memory in rat pups (Li XJ et al. 2013; Li Y et al. 2009). These experimental findings suggest that early-life exposure to certain phthalates might have adverse effects on behavior, learning, and memory.

Potential long-term effects of phthalate exposure during the fetal period on the behavioral profile of school-age children are uncertain. We conducted a 9-year follow-up study to estimate associations of various prenatal phthalates with distinct behavioral syndromes including internalizing and externalizing problem scores in 8-year-old children in Taiwan.

## Methods

**Figure 1:**
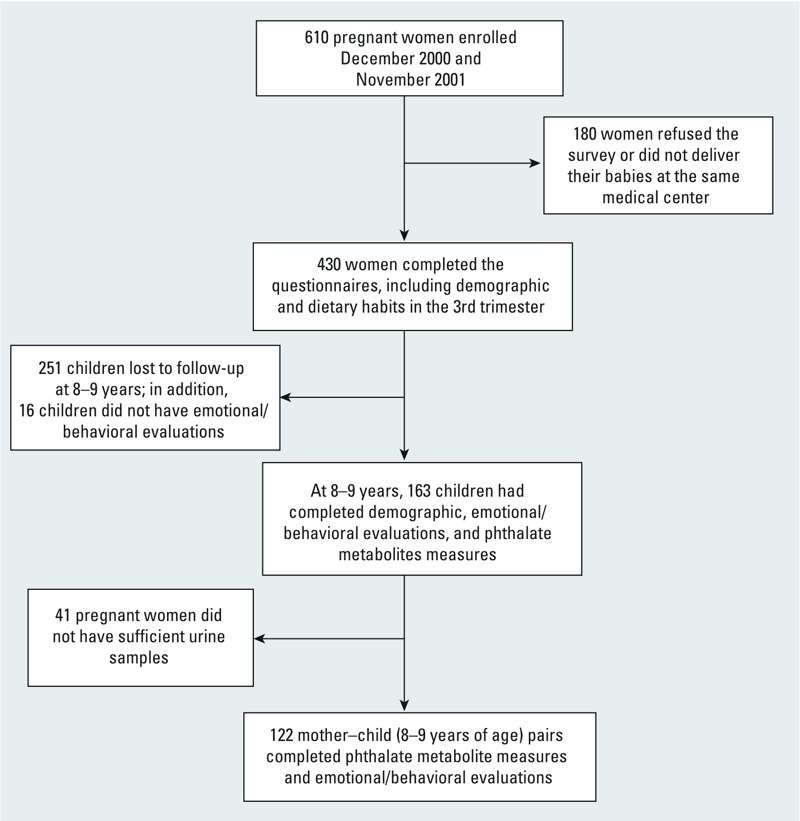
The follow-up diagram of the study population from pregnancy to child’s age of 8–9 years.

*Study population and recruitment*. This was a birth cohort study examining multiple prenatal and postnatal factors in relation to child health outcomes, part of the nationwide Taiwan Maternal and Infant Cohort Study. (Lin LC et al. 2011; Wang et al. 2005). The research profile is depicted in Figure 1. The study participants were pregnant women without clinical complications of eclampsia or preeclampsia, who were 25–34 years of age and delivered their babies in a medical center located in central Taiwan between 1 December 2000 and 30 November 2001 (*n* = 610). The recruitment and follow-up protocols were both approved by the Institutional Review Board of the National Health Research Institutes in Taiwan. After receiving detailed explanations of the study, each of the pregnant women and their children at 8 years of age provided informed consent for the questionnaire interview, sample collections, and behavioral evaluations. A total of 430 pregnant women in the third trimester were recruited and invited to provide urine samples and information regarding demographics, dietary habits, and reproductive and medical histories. Urine samples were collected during the third trimester (*n* = 388).

Follow-up was done when the children were 8 years old in order to evaluate these children’s urinary phthalate metabolite levels as well as their behavioral problems. Of all of those first enrolled in the study center, 251 children were lost to follow-up at 8–9 years mainly because they moved away. An additional 16 caregivers did not complete behavioral evaluations for their children. For a total of 163 8-year-old children, we had completed demographic questionnaires (including parental education levels, parental occupation, family income, parity) and behavioral evaluations. We also assessed Full-Scale IQ scores at 8–9 years of age using the Chinese version of the Wechsler Intelligence Scale for Children-version III (WISC-III) (Wechsler 1991). Because urine samples collected from 41 of the pregnant women during the third trimester were not sufficient for phthalate metabolite analyses, we finally had 122 pairs of women and their children with both urine phthalate metabolite levels and behavioral evaluations.

*CBCL*. Mothers completed the CBCL at the visit when their children were 8 years old. The mothers’ evaluations were based on the status of their children in the preceding 6 months. The CBCL/4-18 (Achenbach 1991) comprised 20 competence items and 118 behavioral/emotional items with a 3-point rating scale (0 if not true, 1 if somewhat true or sometimes true, and 2 if very true or often true). On the basis of cross-informant analysis, eight narrow-band behavioral syndromes (i.e., Withdrawn, Somatic Complains, Anxious/Depressed, Social Problems, Thought Problems, Attention Problems, Delinquent Behavior, and Aggressive Behavior) were defined according to Achenbach (1991). Some syndromes were further summed to provide scores for broad-band Internalizing Problems (the sum of Withdrawn, Somatic Complaints, and Anxious/Depressed) and Externalizing Problems (the sum of Delinquent Behavior and Aggressive Behavior). The reliability and validity of the Chinese version of the CBCL (CBCL-C) were satisfactory for Taiwanese adolescents (Yang et al. 2000). A recent study further indicated that the CBCL has cross-cultural generalizability in as many as 23 societies, including Taiwan (Ivanova et al. 2007). T-scores of the CBCL were derived from well-established normative data in Taiwan (Yang et al. 2000). A T-score of 50 represents the mean of the normative group with the SD of 10. Most of the means (± SDs) for the CBCL T-scores among the 122 children in this study were similar to the population norm of Taiwanese children, which were 45.36 ± 6.85 for Withdrawn, 48.26 ± 8.64 for Somatic Complaints, 49.94 ± 9.05 for Anxious/Depressed, 51.91 ± 9.72 for Social Problems, 47.65 ± 9.83 for Thought Problems, 47.98 ± 8.55 for Attention Problems, 49.39 ± 8.25 for Delinquent Behavior, 50.27 ± 9.85 for Aggressive Behavior, 47.83 ± 8.24 for Internalizing Problems, and 50.01 ± 9.33 for Externalizing Problems.

*Phthalate measurements*. Seven phthalate metabolites—mono-methyl phthalate (MMP), mono-ethyl phthalate (MEP), mono-butyl phthalate (MBP), mono-benzyl phthalate (MBzP), the metabolites of DMP, DEP, DBP, and BBzP, respectively; and mono-2-ethylhexyl phthalate (MEHP), mono-2-ethyl-5-hydroxyhexyl phthalate (MEHHP), mono-2-ethyl-5-oxohexyl phthalate (MEOHP), the metabolites of DEHP, representing exposures to the five commonly used phthalates—were measured in maternal urine samples collected during the third trimester of pregnancy, and children’s urine samples collected at 8–9 years of age. Details of the analysis and quality assurance and control procedures were described previously (Lin LC et al. 2011). In brief, we used β-glucuronidase enzymatic deconjugation of the metabolites from their glucuronidated form, followed by on-line solid-phase extraction (SPE) and quantification using liquid chromatography–electrospray ionization–tandem mass spectrometry. The intra-day variations were < 10%, and intra-day recoveries were at 100 ± 20% at three different concentrations, 25%, 50%, and 75%, of the individual substances. The limits of detection (LOD) for MMP, MEP, MBP, MBzP, MEHP, MEHHP, and MEOHP were 3.4, 2.2, 1.6, 0.99, 0.55, 0.23, and 0.26 ng/mL, respectively. When phthalate metabolite levels were below the LOD, we replaced these values with 50% of the LOD values. Urinary creatinine level was measured by the spectrophotometric method, with picric acid as reactive, and read at 520 nm at the central laboratory of Kaohsiung Medical University Chung-Ho Memorial Hospital.

*Statistical analyses*. We used chi-square tests and independent *t*-tests to compare the differences in demographic characteristics between pregnant women with children whom we followed and those pregnant women lost to follow-up, and Mann–Whitney *U*-tests to compare differences in phthalate metabolite levels between these two groups of pregnant women.

The distributions of studied phthalate metabolites were skewed to the right and thus were log_10_-transformed to fit the model needs. With 122 mother–child pairs followed, we could detect correlations of ≥ 0.25 between the phthalate metabolites and behavior outcomes with 80% power and 5% type 1 error. The T-scores of the CBCL syndromes were approximately normally distributed. The CBCL scales can be analyzed as continuous scores, or the children can be classified as the normal, borderline, or clinical range (Achenbach 1991; Yang et al. 2000). According to the percentiles in the normative population in Taiwan (Yang et al. 2000), T-scores above the 95th percentile represented the clinical range, and T-scores that ranged from the 90th to the 95th percentile fall into the borderline range. Multivariable linear regression for continuous outcomes were used to estimate associations between prenatal or children’s urine phthalate levels and children’s behavioral problem scores at 8–9 years of age. Meanwhile, polytomous logistic regression models were used to estimate odds ratios (ORs) comparing exposures among those classified as having borderline (90–95th percentile) behavior with those with scores < 90th percentile, and ORs comparing exposures among those classified as having clinical (> 95th percentile) behavior with those with scores < 90th percentile in the model simultaneously. ORs for borderline/clinical combined were estimated using dichotomous logistic regression. Regarding possible sex difference in the beta estimates, we used independent *t*-tests to assess the difference in CBCL score by child sex, and a Wald test to examine the significance level of the cross-product term in the following model: Y = α + β1 sex + β2 log_10_ (creatinine-corrected phthalates) + β3 sex × log_10_ (creatinine-corrected phthalates). From a pool of covariates that were possibly associated with the phthalate concentrations or CBCL scores in previous studies (Fan et al. 2013; Raadal et al. 1994; Tehrani-Doost et al. 2011), those remaining significantly correlated (*p* < 0.05) with at least one of the behavioral/emotional problems on the CBCL were selected as covariates in regression models. Model covariates for CBCL outcomes were children’s sex, IQ, and family income. In this study, children’s parental education was not controlled for because it was not significantly associated with either phthalate concentration or CBCL scores. Adjusting for children’s IQ did not influence coefficients for exposure–outcome associations when models were also adjusted for sex and family income. Analyses were performed with SAS 9.1 for Windows (SAS Institute Inc., Cary, NC, USA). All tests were two-tailed, and significance was set at *p* < 0.05 unless otherwise specified.

## Results

*Cohort characteristics*. Demographic characteristics of women with children who were followed (*n* = 122) and those lost to follow-up (*n* = 266) are shown in Table 1. No significant differences in family income or maternal smoking during pregnancy were observed between the groups. Pregnant women who were followed had a greater proportion of higher levels of education (i.e., junior college or university) than those lost to follow-up. The distribution of seven urinary phthalate metabolites adjusted for creatinine in the two groups is shown in Table 2. Similar concentrations of all phthalate levels were found. We also observed that boys had higher average scores than girls on Attention (mean ± SD: 49.4 ± 9.3 for boys vs. 46.3 ± 7.7 for girls) and Aggressive Behaviors (52.5 ± 10.5 for boys vs. 48.9 ± 9.0 for girls), and Externalizing Problems (52.2 ± 10.2 for boys vs. 48.7 ± 8.3 for girls) (see Supplemental Material, Table S1). However, there appeared to be no significant sex difference in the beta estimates (see Supplemental Material, Table S2). We plotted the log_10_-transformed maternal creatinine-corrected MEOHP levels corresponding to externalizing problem scores. The parallel regression lines for boys and girls confirmed the similar pattern of beta estimates between sexes (see Supplemental Material, Figure S1). Thus the combined data for boys and girls were analyzed and presented to produce reasonably precise estimates of association.

**Table 1:** Demographic characteristics of the pregnant women recruited in 2000–2001 in central Taiwan.

Parental variable	Pregnant women lost to follow-up^*a*^	Pregnant women who were followed	*p*-Value
*n*	266	122
Age at delivery (years)	28.61 ± 4.47	29.28 ± 3.69	0.153
< 35	222 (93.3)	115 (94.3)
≥ 35	16 (6.7)	7 (5.7)
Maternal education			0.003
≤ High school	132 (53.2)	42 (34.4)
Junior college	83 (33.5)	57 (46.7)
≥ University	33 (13.3)	23 (18.9)
Paternal education			0.040
≤ High school	119 (49.8)	44 (36.1)
Junior college	71 (29.7)	49 (40.2)
≥ University	49 (20.5)	29 (23.7)
Family income per year			0.106
< US$20,000	111 (47.2)	43 (35.8)
US$20,000–50,000	120 (51.1)	74 (61.7)
> US$50,000	4 (1.7)	3 (2.5)
Maternal smoking during pregnancy			0.668
Yes	5 (2.1)	1 (0.8)
No	235 (97.9)	121 (99.2)
Values are *n* (%) or mean ± SD. *p*-Values indicate differences between groups using the *t*-test for continuous variables and chi-square or Fisher’s exact test for categorical variables.^***a***^One woman gave birth to two babies between 1 December 2000 and 30 November 2001.			

**Table 2:** Geometric mean (GM) of urinary phthalate metabolite concentrations (μg/g creatinine) in pregnant women.

Phthalate metabolite	Percent > LOD	Pregnant women lost to follow-up (*n *= 266)^*a*^		Pregnant women who were followed (*n *= 122)		*p*‑Value
		Mean ± SD	GM (95% CI)	Mean (SD)	GM (95% CI)	
MMP	94	80.70 ± 75.77	54.13 (48.13, 60.88)	87.30 ± 98.30	54.53 (44.95, 66.14)	0.896
MEP	100	103.2 ± 158.0	65.09 (58.59, 72.31)	99.87 ± 134.8	61.71 (52.45, 72.60)	0.535
MBP	100	124.5 ± 187.0	74.28 (66.40, 83.09)	109.1 ± 121.6	66.88 (55.93, 79.99)	0.442
MBzP	99	25.06 ± 22.80	18.61 (16.98, 20.39)	20.20 ± 16.07	15.88 (13.87, 18.19)	0.103
MEOHP	79	30.48 ± 72.46	9.50 (7.71, 11.71)	39.18 ± 99.65	13.59 (10.27, 18.00)	0.104
MEHHP	89	24.93 ± 51.22	6.61 (5.25, 8.33)	26.77 ± 57.70	7.91 (5.69, 11.02)	0.538
MEHP	99	34.36 ± 48.73	20.02 (17.81, 22.51)	30.06 ± 67.63	16.93 (14.32, 20.02)	0.134
*p-*Values for the difference between pregnant women lost to follow-up and pregnant women who were followed for analysis were estimated using the Mann–Whitney *U*-test. DEHP metabolites: MEHP, MEHHP, MEOHP.^***a***^One woman gave birth to two babies between 1 December 2000 and 30 November 2001.						

*Phthalates and CBCL*. Table 3 shows the multivariable linear regression analysis results of log_10_ creatinine-corrected concentrations of prenatal phthalate metabolites and children’s behavior scores at 8–9 years of age. When adjusted for sex, IQ, and family income, both MBP and MEOHP exhibited average difference in scores associated with a 1-unit increase in log_10_-transformed creatinine-corrected urine concentrations for Delinquent Behavior [β = 3.57, 95% confidence interval (CI): 0.20, 6.94 for MBP; β = 3.95, 95% CI: 1.81, 6.09 for MEOHP], Aggressive Behavior (β = 4.21, 95% CI: 0.31, 8.11 for MBP; β = 3.32, 95% CI: 0.77, 5.87 for MEOHP), and Externalizing Problems (i.e., sum of Delinquent Behavior and Aggressive Behavior) (β = 4.29, 95% CI: 0.59, 7.59 for MBP). In addition, MEHHP was associated with scores for Delinquent Behavior (β = 1.87, 95% CI: 0.05, 3.69), and MEHP was associated with scores for Delinquent Behavior (β = 3.77, 95% CI: 0.16, 7.38) and Externalizing Problems (β = 4.28, 95% CI: 0.03, 8.26). Other than externalizing domain behavior problem scores, MEOHP was associated with increased scores for Social Problems (β = 2.98; 95% CI: 0.37, 5.59). Nevertheless, none of the maternal phthalate metabolite concentrations were associated with children’s scores in internalizing domain behavior problems (Somatic Complaints, Anxious/Depressed, or Internalizing Problem scores) (all *p-*values > 0.05). There were no significant cross-sectional associations between any children’s urinary creatinine-corrected phthalates metabolite concentrations measure at 8–9 years of age and their behavior problem scores (all *p*-values > 0.05 based on all the 122 children; data not shown).

**Table 3:** Regression coefficients (95% CIs) for 1-unit increase in log_10_-transformed creatinine-corrected concentrations of maternal urinary phthalate metabolites on CBCL scores in 8-year-old children (*n* = 122).

Behavior/metabolite (log_10_)	MMP	MEP	MBP	MBzP	MEOHP	MEHHP	MEHP
Without adjusting for covariates
Withdrawn	–0.42 (–3.07, 2.23)	–2.67 (–5.77, 0.43)	2.72 (–0.08, 5.52)	–1.54 (–5.28, 2.20)	0.94 (–0.86, 2.74)	0.04 (–1.51, 1.59)	1.62 (–1.42, 4.66)
Somatic Complaints	–1.52 (–4.81, 1.77)	–1.29 (–5.21, 2.63)	1.53 (–2.02, 5.08)	–1.95 (–6.63, 2.73)	1.86 (–0.39, 4.11)	0.84 (–1.08, 2.76)	1.52 (–2.28, 5.32)
Anxious/Depressed	–1.14 (–4.49, 2.21)	–1.12 (–5.12, 2.88)	2.93 (–0.66, 6.52)	–1.24 (–6.02, 3.54)	1.69 (–0.60, 3.98)	1.39 (–0.55, 3.33)	1.13 (–2.73, 4.99)
Social Problems	0.10 (–3.57, 3.77)	–3.53 (–7.82, 0.76)	3.87 (–0.01, 7.75)	0.35 (–4.84, 5.54)	3.62 (1.19, 6.05)**	2.36 (0.26, 4.46)*	2.10 (–2.09, 6.29)
Thought Problems	–2.75 (–6.49, 0.99)	0.94 (–3.55, 5.43)	0.50 (–3.58, 4.58)	–1.56 (–6.93, 3.81)	–0.09 (–2.29, 2.11)	–0.08 (–2.28, 2.12)	3.56 (–0.75, 7.87)
Attention Problems	–0.05 (–3.34, 3.24)	–2.82 (–6.70, 1.06)	1.21 (–2.34, 4.76)	–2.43 (–7.09, 2.23)	1.92 (–0.31, 4.15)	0.51 (–1.41, 2.43)	1.42 (–2.36, 5.20)
Delinquent Behavior	–0.22 (–3.40, 2.96)	0.26 (–3.50, 4.02)	3.87 (0.52, 7.22)*	–0.40 (–4.91, 4.11)	4.50 (2.46, 6.54)**	2.57 (0.77, 4.37)**	3.71 (0.10, 7.32)*
Aggressive Behavior	0.70 (–2.98, 4.38)	–1.67 (–6.04, 2.70)	4.32 (0.40, 8.24)*	–0.81 (–6.06, 4.44)	3.94 (1.49, 6.39)**	1.98 (–0.14, 4.10)	4.13 (–0.06, 8.32)
Internalizing Problems	–1.20 (–4.32, 1.92)	–1.90 (–5.58, 1.78)	2.92 (–0.41, 6.25)	–1.77 (–6.20, 2.66)	1.77 (–0.35, 3.89)	1.77 (–0.35, 3.89)	1.61 (–1.98, 5.20)
Externalizing Problems	0.45 (–3.08, 3.98)	–1.17 (–5.36, 3.02)	4.47 (0.75, 8.19)*	–0.74 (–5.78, 4.30)	4.38 (2.07, 6.69)**	2.30 (0.28, 4.32)*	4.28 (0.28, 8.28)*
Adjusting for children’s IQ, sex, and family income
Withdrawn	–0.92 (–3.61, 1.77)	–2.50 (–5.62, 0.62)	2.31 (–0.53, 5.15)	–2.21 (–6.01, 1.59)	0.28 (–1.62, 2.18)	–0.49 (–2.06, 1.08)	1.59 (–1.49, 4.67)
Somatic Complaints	–2.00 (–5.41, 1.41)	–1.22 (–5.22, 2.78)	1.02 (–2.65, 4.69)	–2.38 (–7.26, 2.50)	1.46 (–0.95, 3.87)	0.45 (–1.55, 2.45)	1.42 (–2.50, 5.34)
Anxious/Depressed	–1.48 (–4.91, 1.95)	–1.00 (–5.02, 3.02)	2.06 (–1.61, 5.73)	–2.67 (–7.53, 2.19)	0.92 (–1.49, 3.33)	0.88 (–1.12, 2.88)	1.32 (–2.60, 5.24)
Social Problems	–0.67 (–4.45, 3.11)	–3.37 (–7.74, 1.00)	3.31 (–0.69, 7.31)	–0.76 (–6.13, 4.61)	2.98 (0.37, 5.59)*	1.83 (–0.35, 4.01)	1.90 (–2.41, 6.21)
Thought Problems	–3.61 (–7.49, 0.27)	1.42 (–3.17, 6.01)	0.16 (–4.05, 4.37)	–1.73 (–7.34, 3.88)	–0.64 (–3.42, 2.14)	–0.80 (–3.09, 1.49)	3.73 (–0.74, 8.20)
Attention Problems	–1.30 (–4.49, 1.89)	–2.00 (–5.70, 1.70)	0.78 (–2.63, 4.19)	–3.46 (–7.97, 1.05)	0.83 (–1.42, 3.08)	–0.50 (–2.36, 1.36)	1.35 (–2.30, 5.00)
Delinquent Behavior	–0.99 (–4.18, 2.20)	0.65 (–3.09, 4.39)	3.57 (0.20, 6.94)*	–0.26 (–4.81, 4.29)	3.95 (1.81, 6.09)**	1.87 (0.05, 3.69)*	3.77 (0.16, 7.38)*
Aggressive Behavior	–0.49 (–4.21, 3.23)	–0.92 (–5.27, 3.43)	4.21 (0.31, 8.11)*	–1.39 (–6.68, 3.90)	3.32 (0.77, 5.87)*	1.20 (–0.96, 3.36)	4.11 (–0.08, 8.30)
Internalizing Problems	–1.70 (–4.86, 1.46)	–1.75 (–5.45,1.95)	2.17 (–1.20, 5.54)	–2.87 (–7.36, 1.62)	1.02 (–1.21, 3.25)	0.42 (–1.42, 2.26)	1.67 (–1.96, 5.30)
Externalizing Problems	–0.68 (–4.23, 2.87)	–0.49 (–4.63, 3.65)	4.29 (0.59, 7.99)*	–1.13 (–6.17, 3.91)	3.74 (1.33, 6.15)**	1.49 (–0.55, 3.53)	4.28 (0.03, 8.26)*
DEHP metabolites: MEHP, MEHHP, MEOHP. *p < 0.05. **p < 0.01.							

Higher prenatal phthalate metabolite concentrations were associated with higher ORs of borderline or clinical range scores on the CBCL externalizing domain behaviors (Table 4). Given the very small numbers in the borderline or clinical range of the CBCL scores, we thus further merged the borderline and the clinical groups into a single group (borderline/clinical) to improve statistical power and precision. ORs represent a 1-unit increase on log_10_-transformed creatinine-corrected maternal urine concentrations. Four of the seven studied phthalate metabolites exhibited increased ORs on the borderline/clinical ranges of Externalizing Problems (14 cases total): MBP (OR = 8.37; 95% CI: 1.61, 43.45), MEOHP (OR = 16.68; 95% CI: 3.08, 90.18), MEHHP (OR = 3.10; 95% CI: 1.09, 8.84), and MEHP (OR = 22.97; 95% CI: 3.34, 158.06). Among these four phthalate metabolites, only MEOHP exhibited increased ORs on the borderline/clinical ranges of Aggressive Behavior (OR = 3.22; 95% CI: 1.12, 9.28 based on 16 cases). Besides, MEP was associated with borderline range, but not clinical range, for Delinquent Behavior (OR = 7.15; 95% CI: 1.26, 40.52, based on 8 cases), and with clinical range on Externalizing Problems (OR = 6.09; 95% CI: 1.01, 36.74, based on 8 cases). With regard to the associations between children’s phthalate metabolites and children’s behavior problems in terms of borderline/clinical range at 8–9 years of age, only one significant association was implicated (data not shown). There was an association between MMP and scores on the borderline/clinical range of Withdrawn (OR = 9.15; 95% CI: 1.34, 62.64, based on 5 cases). The inclusion or exclusion of child’s IQ did not alter the observed patterns of association (data not shown).

**Table 4:** ORs (95% CIs) for CBCL scores in the borderline and/or clinical compared with normal range on the externalizing behaviors for each log10-unit increase in creatinine-corrected concentrations of maternal urinary phthalate metabolites (*n* = 122).

Behavior/metabolite (log_10_)	Borderline	Clinical	Borderline/clinical
Delinquent Behavior
No. of observations	*n* = 8	*n* = 6	*n* = 14
MMP	2.07 (0.33, 13.07)	1.14 (0.17, 7.43)	1.55 (0.40, 6.06)
MEP	7.15 (1.26, 40.52)*	1.06 (0.12, 9.75)	3.33 (0.85, 13.00)
MBP	5.34 (0.74, 38.29)	7.28 (0.84, 63.17)	6.17 (1.34, 28.43)*
MBzP	9.99 (0.52, 190.64)	4.83 (0.22, 108.02)	6.94 (0.75, 64.12)
MEOHP	27.31 (3.22, 231.79)**	22.91 (2.32, 226.13)**	24.99 (4.17, 149.65)**
MEHHP	2.62 (0.77, 8.95)	4.21 (0.80, 22.32)	3.11 (1.11, 8.72)*
MEHP	74.04 (4.60, > 999.99)**	7.00 (0.65, 75.77)	21.68 (3.41, 137.60)**
Aggressive Behavior
No. of observations	*n* = 7	*n* = 9	*n* = 16
MMP	0.86 (0.16, 4.54)	1.55 (0.25, 9.51)	1.09 (0.31, 3.81)
MEP	0.43 (0.04, 4.18)	4.19 (0.75, 23.51)	1.58 (0.41, 6.16)
MBP	0.25 (0.03, 2.18)	7.18 (1.05, 49.41)*	1.65 (0.44, 6.28)
MBzP	0.90 (0.09, 9.03)	0.94 (0.09, 9.59)	0.89 (0.17, 4.71)
MEOHP	1.95 (0.53, 7.13)	6.88 (1.30, 36.34)*	3.22 (1.12, 9.28)*
MEHHP	0.74 (0.29, 1.92)	4.99 (1.06, 23.54)*	1.49 (0.67, 3.29)
MEHP	0.89 (0.11, 6.96)	9.77 (1.50, 63.70)*	2.92 (0.77, 11.12)
Externalizing Problems
No. of observations	*n* = 6	*n* = 8	*n* = 14
MMP	2.60 (0.30, 22.27)	0.96 (0.18, 5.28)	1.42 (0.35, 5.75)
MEP	0.51 (0.05, 5.64)	6.09 (1.01, 36.74)*	2.20 (0.54, 9.00)
MBP	38.64 (2.31, 646.34)*	3.63 (0.53, 24.81)	8.37 (1.61, 43.45)*
MBzP	0.53 (0.06, 4.82)	6.67 (0.36, 124.50)	1.69 (0.22, 12.81)
MEOHP	10.37 (1.40, 76.54)*	28.49 (3.16, 256.45)**	16.68 (3.08, 90.18)**
MEHHP	1.70 (0.50, 5.80)	7.41 (1.26, 43.38)*	3.10 (1.09, 8.84)*
MEHP	16.42 (1.70, 158.92)*	31.01 (3.01, 319.35)**	22.97 (3.34, 158.06)**
Models were controlled for children’s sex, IQ, and family income. DEHP metabolites: MEHP, MEHHP, MEOHP. T-scores of the CBCL were derived from well-established normative data in Taiwan (Yang et al. 2000). Polytomous logistic regression models were used to estimate ORs comparing exposures among those classified as borderline (90–95th percentile) behavior to those with scores < 90th percentile, and ORs comparing exposures among those classified as clinical (> 95th percentile) behavior to those with scores < 90th percentile in the model simultaneously. ORs for borderline/clinical combined were estimated using dichotomous logistic regression. *p < 0.05. **p < 0.01.			

## Discussion

We estimated positive associations between creatinine-corrected maternal urinary concentrations of DEHP and DBP metabolites and externalizing domain behavior problem scores (i.e., scores for Delinquent and Aggressive Behaviors, and Externalizing Problems) in 8- to 9-year-old children. Engel et al. (2010) reported that Externalizing Problems and Aggressive Behavior scores in 4- to 9-year-old boys were associated with maternal urinary MBP and MEP concentrations in the third trimester. In our study population, boys and girls combined, maternal urine MBP concentrations were associated with higher average scores and with scores in the borderline/clinical combined range for Externalizing Problems, and maternal MEP concentrations were associated with Externalizing Problems scores in the clinical range. Whyatt et al. (2012) reported that maternal urine MnBP concentrations were positively associated with CBCL Internalizing Behavior scores at 3 years of age. We observed a positive but nonsignificant association between MBP (representing combined MnBP and MiBP concentrations) and Internalizing Behavior scores at 8–9 years of age in our study population. In addition, we estimated a significant positive association between maternal urine concentrations of MEOHP (a DEHP metabolite) and Social Problems scores. To our knowledge, this has not been reported previously.

A biological mechanism that might result in negative effects of phthalates on brain development and behavior has not been established. It has been proposed that DEHP might impair fetal brain development through activation of Trim17 protein via PPARγ (peroxisome proliferator-activated receptor-γ) leading to apoptosis based on an *in vitro* study of neuroblastoma cells (Lin CH et al. 2011). Chen TA et al. (2011) proposed that MEHP might disturb neurodevelopment by suppressing cell proliferation and promoting cell differentiation towards a cholinergic phenotype that may relate to hyperactivity based on *in vitro* findings in neuronotypic PC12 cells. In rat pups, perinatal exposure to DBP induced cell apoptosis in hippocampal neurons and appeared to impair the spatial learning and memory (Li XJ et al. 2013; Li Y et al. 2009). In addition, it has been suggested that phthalates might indirectly affect neurodevelopment through effects on essential fatty acids or thyroid function. Xu et al. (2008) reported that *in utero* exposure to DEHP influenced the transfer of essential fatty acids across the placenta and was associated with altered lipid content in the rat fetal brain. In a rat thyroid cell line, DEHP and DBP appeared to affect iodide uptake activity through modulation of sodium/iodide symporter (Wenzel et al. 2005). In a study of 76 women during the second trimester of pregnancy, urine concentrations of the DBP metabolite MBP were associated with significantly lower serum free thyroxine (fT4) concentrations (Huang et al. 2007). Furthermore, adverse effects of maternal thyroid dysfunction on neurodevelopment or early postnatal brain development were well known (Haddow et al. 1999; Porterfield and Hendrich 1993). Thus DBP and DEHP might affect growth and development of central nervous system in both direct and indirect modes.

*In utero* exposure assessment for the present study was limited to a single spot urine sample collected in the third trimester of pregnancy. A previous report (Morreale de Escobar et al. 2004) suggested that brain development begins at early conception, which might be the most vulnerable time in neurodevelopment. We assumed that a single urine concentration represented typical exposure, and that sources or exposure patterns were consistent during pregnancy. However, Braun et al. (2012) reported relatively high temporal variability of DEHP metabolites in pregnant women. Nondifferential exposure misclassification might attenuate effect estimates leading to underestimated associations between prenatal phthalate and behavior problems.

The attrition rate was high in the current study. We examined differences between the lost and followed groups and found that all studied characteristics and phthalate concentrations were similar, except for higher parental educational level in the followed group. In the future, larger sample sizes might help the situations and also offer the opportunity of further stratification by sex. In addition, we were not able to adjust for other correlated exposures, such as bisphenol A, in the current study. Last, we did not distinguish between the two isomers of MnBP and mono-isobutyl phthalate (MiBP). We found that MnBP occupies the majority of MBP for about 81 ± 10% in several of our participants (*n* = 99). This is consistent with the previous study (Whyatt et al. 2012). Future prospective studies with sufficient sample size will be needed to confirm the sensitive susceptibility to phthalates during pregnancy, and to evaluate sex-specific associations between phthalates and the risk of behavior problems with consideration of phthalate isomers and co-exposure to other endocrine disruptors in children.

## Conclusions

Our data supported positive associations between maternal DBP and DEHP metabolite concentrations measured in the third-trimester urine samples and children’s externalizing domain problem scores assessed at 8–9 years of age. The results suggest that prenatal exposure to such chemicals might play a role in the development of behavioral problems later in the children’s lives.

## Supplemental Material

(314 KB) PDF
