# Supplementary material for: Prenatal Exposure to Phthalate Esters and Behavioral Syndromes in Children at 8 Years of Age: Taiwan Maternal and Infant Cohort Study
Source: Environ Health Perspect. 2014 Oct 3;123(1):95–100. doi: 10.1289/ehp.1307154 (PMC4286269; doi:10.1289/ehp.1307154)
Supplement: (314 KB) PDF [file ehp.1307154.s001.508.pdf]

Environmental Health Perspectives doi:10.1289/ehp.1307154

Supplemental Material

Prenatal Exposure to Phthalate Esters and Behavioral Syndromes in Children at Eight Years of Age: Taiwan Maternal and Infant Cohort Study

Yin-Ju Lien, Hsiu-Ying Ku, Pen-Hua Su, Suh-Jen Chen, Hsiao-Yen Chen, Pao-Chi Liao, Wei-J. Chen, and Shu-Li Wang

Table S1. Regression coefficients (with 95% confidence intervals) of Children Behavior Checklist (CBCL) scores on log_{10}-transformed creatinine-corrected maternal urinary concentrations in 8-year-old children by gender (n=122).

Behavior/metabolite	Mean ± SD[b]	MMP	MEP	MBP	MBzP	MEOHP	MEHHP	MEHP
Boys (n=59)[a]								
Withdrawn	44.7 ± 6.8	-1.92 (-5.68, 1.83)	-2.02 (-6.58, 2.55)	-1.61 (-6.37, 3.14)	-3.37 (-8.47, 1.72)	-0.20 (-2.78, 2.38)	-1.29 (-3.60, 1.01)	-0.45 (-4.91, 4.02)
Somatic complains	47.6 ± 7.9	-2.06 (-6.41, 2.30)	1.11 (-4.22, 6.43)	3.06 (-2.41, 8.53)	0.59 (-5.41, 6.59)	2.21 (-0.72, 5.14)	0.67 (-2.04, 3.37)	3.31 (-1.79, 8.40)
Anxious/depressed	48.9 ± 7.8	-2.90 (-7.33, 1.53)	0.38 (-5.09, 5.84)	-0.64 (-6.30, 5.03)	-3.26 (-9.35, 2.82)	1.40 (-1.64, 4.44)	0.53 (-2.24, 3.30)	-0.63 (-5.93, 4.67)
Social problems	51.4 ± 9.8	-2.19 (-7.55, 3.18)	-1.31 (-7.86, 5.23)	2.73 (-4.03, 9.48)	-0.30 (-7.68, 7.08)	3.89 (0.38, 7.41)*	2.33 (-0.93, 5.60)	3.82 (-2.46, 10.09)
Thought problems	48.4 ± 12	-3.42 (-10.47, 3.62)	7.47 (-0.91, 15.85)	0.31 (-8.64, 9.27)	-0.89 (-10.60, 8.83)	0.82 (-4.02, 5.66)	0.26 (-4.12, 4.65)	4.30 (-4.00, 12.59)
Attention problems	49.4 ± 9.3*	-3.47 (-8.65, 1.71)	1.06 (-5.33, 7.45)	-4.04 (-10.58, 2.50)	-3.20 (-10.34, 3.95)	1.29 (-2.28, 4.86)	-0.79 (-4.03, 2.45)	1.23 (-4.97, 7.43)
Delinquent behavior	50.8 ± 8.9	-3.31 (-8.20, 1.59)	0.35 (-5.69, 6.40)	1.53 (-4.72, 7.79)	-2.28 (-9.05, 4.50)	5.04 (1.96, 8.13)*	2.74 (-0.24, 5.71)	4.66 (-1.06, 10.39)
Aggressive behavior	52.5 ± 10.5*	-5.55 (-11.07, -0.04)*	-2.28 (-9.20, 4.63)	-3.97 (-11.10, 3.15)	-4.68 (-12.40, 3.03)	2.85 (-0.97, 6.67)	0.12 (-3.41, 3.65)	0.46 (-6.28, 7.21)
Internalizing problems	46.9 ± 7.6	-2.79 (-6.83, 1.25)	-0.16 (-5.16, 4.84)	0.06 (-5.12, 5.25)	-2.66 (-8.23, 2.92)	1.33 (-1.45, 4.11)	0.03 (-2.51, 2.57)	0.55 (-4.30, 5.39)
Externalizing problems	52.2 ± 10.2*	-5.22 (-10.62, 0.18)	-1.61 (-8.38, 5.16)	-2.51 (-9.51, 4.50)	-4.24 (-11.79,3.31)	3.73 (0.07, 7.39)*	0.95 (-2.49, 4.39)	1.81 (-4.75, 8.38)
Girls (n=63)[a]								
Withdrawn	45.9 ± 7.0	0.36 (-3.73, 4.45)	-2.63 (-7.20, 1.94)	4.74 (1.02, 8.46)*	-0.34 (-6.47, 5.79)	0.88 (-2.06, 3.82)	0.35 (-1.95, 2.65)	3.76 (-0.76, 8.27)
Somatic complains	48.0 ± 8.6	-1.22 (-6.59, 4.16)	-2.56 (-8.61, 3.48)	-0.42 (-5.58, 4.75)	-4.68 (-12.65, 3.30)	0.77 (-3.11, 4.65)	0.63 (-2.39, 3.65)	-0.26 (-6.34, 5.83)
Anxious/depressed	50.0 ± 9.5	-0.18 (-5.61, 5.25)	-2.43 (-8.53, 3.67)	2.95 (-2.20, 8.11)	-2.37 (-10.49, 5.75)	0.35 (-3.57, 4.27)	0.98 (-2.06, 4.02)	2.32 (-3.78, 8.43)
Social problems	51.1 ± 9.3	1.22 (-4.40, 6.83)	-4.93 (-11.14, 1.29)	3.37 (-1.95, 8.69)	-0.69 (-9.11, 7.72)	2.04 (-1.98, 6.06)	1.63 (-1.49, 4.76)	-0.01 (-6.35, 6.34)
Thought problems	46.9 ± 7.0	-3.43 (-7.55, 0.68)	-3.32 (-8.00, 1.36)	-0.43 (-4.48, 3.61)	-2.11 (-8.40, 4.18)	-2.18 (-5.17, 0.80)	-1.60 (-3.93, 0.74)	3.03 (-1.66, 7.72)
Attention problems	46.3 ± 7.7	0.75 (-3.33, 4.83)	-4.84 (-9.27, -0.40)	2.88 (-0.96, 6.73)	-4.15 (-10.18, 1.87)	0.29 (-2.66, 3.24)	-0.51 (-2.80, 1.79)	0.82 (-3.79, 5.43)
Delinquent behavior	48.3 ± 7.6	1.55 (-2.76, 5.87)	1.03 (-3.86, 5.92)	4.25 (0.24, 8.26)*	2.49 (-3.97, 8.95)	2.80 (-0.24, 5.84)	1.27 (-1.14, 3.68)	2.61 (-2.24, 7.46)
Aggressive behavior	48.9 ± 9.0	4.06 (-1.05, 9.16)	-0.32 (-6.21, 5.57)	8.36 (3.87, 12.86)**	1.60 (-6.21, 9.40)	3.73 (0.10, 7.36)*	1.74 (-1.16, 4.63)	6.72 (1.10, 12.34)*
Internalizing problems	48.0 ± 8.4	-0.35 (-5.40, 4.71)	-2.95 (-8.60, 2.70)	3.03 (-1.76, 7.81)	-2.78 (-10.32, 4.75)	0.72 (-2.92, 4.36)	0.82 (-2.01, 3.65)	2.41 (-3.26, 8.08)
Externalizing problems	48.7 ± 8.3	3.54 (-1.19, 8.27)	0.08 (-5.36, 5.53)	7.63 (3.46, 11.80)**	1.99 (-5.22, 9.19)	3.68 (0.35, 7.02)*	1.71 (-0.96, 4.38)	5.88 (0.66, 11.09)*

[a]Adjusted for children's IQ and family income. [b]Independent t test was used to assess the difference in CBCL score by child gender.

DEHP metabolites: MEHP, MEHHP, MEOHP. *P<0.05, **P<0.01.

Table S2. *P*-values for phthalate metabolite level on the behavior scores in boys.[a]

Behavior/metabolite	MMP	MEP	MBP	MBzP	MEOHP	MEHHP	MEHP
Withdrawn	0.30	0.38	0.44	0.19	0.77	0.25	0.96
Somatic complains	0.46	0.97	0.38	0.78	0.25	0.66	0.18
Anxious/depressed	0.32	0.93	0.99	0.43	0.96	0.65	0.65
Social problems	0.47	0.57	0.43	0.99	0.40	0.16	0.22
Thought problems	0.24	<0.05	0.96	0.87	0.66	0.87	0.18
Attention problems	0.20	0.84	0.37	0.42	0.51	0.67	0.57
Delinquent behavior	0.19	0.96	0.08	0.54	0.10	0.50	0.80
Aggressive behavior	0.06	0.52	0.34	0.26	0.17	0.84	0.76
Internalizing problems	0.27	0.82	0.9	0.46	0.43	0.95	0.80
Externalizing problems	0.06	0.60	<0.05	0.28	0.50	0.48	0.46

[a]Wald test was used to examine the significance level of the cross-product term in a model and, $Y = \alpha + \beta 1$ sex + $\beta 2$ $\log_{10}$(creatinine-corrected phthalates) + $\beta 3$ sex × $\log_{10}$(creatinine-corrected phthalates). We used male gender=1 as compared to 0 for female gender.

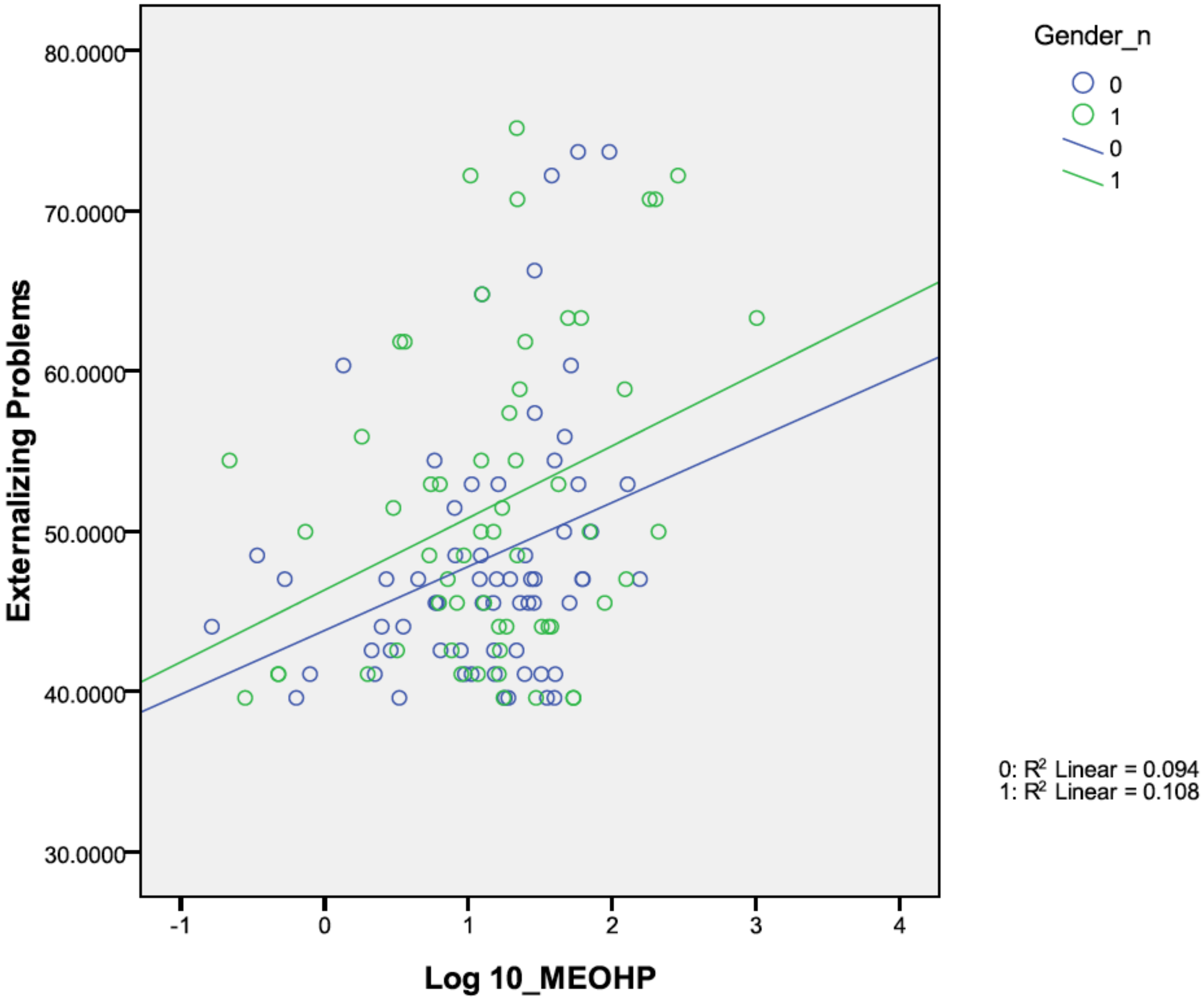

Figure S1. Plot of log $_{10}$-transformed maternal creatinine-corrected MEOHP levels corresponding to externalizing problem scores for boys (green 1) and girls (black 0).
